# Risk Assessment for Venous Thrombosis in Lymphoma and Emerging Biomarkers

**DOI:** 10.3390/ijms27125461

**Published:** 2026-06-17

**Authors:** Alexia Piperidou, Panagiota-Efstathia Nikolaou, Despina Fotiou

**Affiliations:** 1 Department of Hematology and Bone Marrow Transplantation, Medical School, National and Kapodistrian University of Athens, General Hospital of Athens “Laikon”, 11527 Athens, Greece; alexia_piper@hotmail.gr; 2 Laboratory of Pharmacology, School of Pharmacy, National and Kapodistrian University of Athens, 15771 Athens, Greece; nayanik@pharm.uoa.gr; 3 Department of Clinical Therapeutics, School of Medicine, National and Kapodistrian University of Athens, 11527 Athens, Greece; 4 Clinic & Laboratory of Pathophysiology, Department of Medicine, School of Health Sciences, National and Kapodistrian University of Athens, 5 Mikras Asias Street, 11527 Athens, Greece

**Keywords:** biomarkers, CAT, lymphoma, risk, thrombosis

## Abstract

Venous Thrombosis is a frequent and clinically significant complication in lymphoma patients, resulting in increased morbidity, mortality and therapeutic challenges. The pathophysiological mechanisms underlying lymphoma-associated thrombosis are multifactorial, involving patients’ clinical characteristics, tumour biology, systemic inflammation, endothelial dysfunction and therapy-induced prothrombotic changes. Traditional predictive tools for cancer-associated thrombosis (CAT) have shown suboptimal application in lymphoma patients due to disease-specific heterogeneity. The ThroLy score was developed as a lymphoma-specific model incorporating parameters such as extranodal involvement, mediastinal disease, performance status, a prior venous thromboembolic event, and specific laboratory values. While it shows improved predictive value compared with general CAT models, its accuracy remains limited, particularly across different lymphoma subtypes and treatment regimens. Research in the field has therefore focused on evaluating emerging biomarkers—D-dimer, microparticles and inflammatory cytokines—as risk assessment tools. Integrative approaches that combine clinical variables with such biomarkers may yield a more dynamic and individualised risk-prediction model to guide thromboprophylactic strategies. The present review summarises current knowledge on thrombotic risk assessment across lymphoma subtypes and highlights the potential role of novel biomarkers in developing a more precise approach to thrombosis prevention and management. Importantly, it provides a comprehensive overview of currently available literature, highlighting the need for personalised thrombosis risk stratification strategies in lymphoma.

## 1. Introduction

Venous thromboembolism (VTE) represents a frequent and clinically significant complication in patients with lymphoma, contributing substantially to morbidity, mortality, and interruptions in treatment [1,2,3,4]. Although the risk of cancer-associated thrombosis (CAT) has been extensively studied in solid tumours, lymphoma constitutes a distinct clinical entity characterised by marked biological heterogeneity and diverse treatment strategies [4]. Epidemiological data indicate that VTE incidence rate (IR) in lymphoma ranges from approximately 5% to 17%, with higher rates observed in aggressive histologies and during the early phases following diagnosis [1,2,3,4].

The pathogenesis of thrombosis in lymphoma is multifactorial and reflects a complex interplay between tumour-related factors, host characteristics, systemic inflammation, and treatment-related effects. These mechanisms converge on the components of Virchow’s triad, leading to a hypercoagulable state that is both dynamic and context-dependent. Despite this well-recognised risk, current thromboprophylaxis strategies are largely extrapolated from studies in solid tumours, and widely used predictive models, such as the Khorana score, have demonstrated suboptimal performance in lymphoma populations. In addition, lymphoma-specific guidelines for the prophylaxis and treatment of lymphoma-associated thrombosis are missing.

In response to these limitations, lymphoma-specific risk assessment models (RAMs) and novel biomarker-based approaches have been developed to improve risk stratification. However, their clinical utility remains uncertain due to inconsistent validation and limited generalizability. The aim of this review is to summarise current evidence on VTE risk factors and predictive models in lymphoma and to critically evaluate emerging biomarkers that may enable more individualised and dynamic risk assessment strategies. The current management algorithm for the prophylaxis and treatment of thrombosis in patients with lymphoma will also be presented.

## 2. Risk Factors for Thrombosis in Lymphoma

Lymphoma-specific data regarding risk factors for thrombosis are limited. In the following section, we review data from studies published after 2016 to reflect, to the extent possible, more recent practices regarding diagnosis, staging, prognosis and treatment. These studies and older data are included in Table 1.

### 2.1. Epidemiology

Based on a meta-analysis by Caruso et al., which included 18,018 patients across 18 studies, VTE IR in non-Hodgkin Lymphoma (NHL) reaches 6.5% (95% CI, 6.1–6.9%), which is significantly higher than that observed for Hodgkin lymphoma (HL) patients with an IR of 4.7% (95% CI, 3.9–5.6%) [5]. Based on the same study, aggressive lymphomas have the highest IR for events, at 8.3% (95% CI, 7.0–9.9%). The risk is highest at the time of disease diagnosis (47 events/1000 patient-years) and declines sharply over time (7 events/1000 patient-years in the second follow-up year [6]. Overall, among histologic subtypes, a significantly greater risk of VTE was observed in primary mediastinal B-cell lymphoma (PMBCL) and primary CNS lymphoma (PCNSL), with the latter reaching a strikingly high IR of 59.5 [7,8]. Despite these differences in epidemiologic data among studies, which may be explained by differences in reporting, the hypercoagulable state observed not only increases morbidity, but also adversely impacts treatment continuity and overall prognosis in the distinct cancer subgroup of lymphoma patients. The heterogeneity in VTE risk factors within the study population may also contribute to the observed variability.

**Table 1:** Main studies reporting risk factors for thrombosis in Lymphoma.

Study/First Author	Population	Key Risk Factors	VTE Incidence	Type of VTE
Caruso et al. (2010) [5]	Meta-analysis (29 cohorts; *n* = 18,018)	Advanced stage (III–IV), Aggressive histology	Stage III: 6.9%; Stage IV: 11.5%	Venous
Sanfilippo et al. (2016) [9]	NHL (DLBCL + FL; *n* = 2730)	Aggressive histology, BMI ≥ 30, early phase after diagnosis	Highest within first 30 days DLBCL higher vs. FL	Venous
Mahajan et al. (2014) [6]	NHL (*n* = 16,755)	Comorbidities, age (aggressive NHL), gender (low-grade)	Comorbidities increase VTE risk (4-fold), age significant only in high-risk lymphoma	Venous
Santi et al. (2017) [10]	NHL (pooled trials) (*n* = 1717)	Aggressive histology (DLBCL)	DLBCL: ~10–12% (1st year)	More severe VTE episodes in DLBCL
Lund et al. (2015) [11]	Population-based lymphoma cohort (*n* = 10,375)	Advanced stage, CNS Involvement, CVC	CNS involvement increases VTE risk (HR ~ 2.5), treatment period carries highest risk, 6.67-fold increase with CVC	Venous
Goldschmidt et al. (2003) [8]	PCNSL (*n* = 42)	CNS localization	Very high (59.5%)	Not specified (high overall burden)
Borchmann et al. 2019 (GHSG trials) [2]	HL (*n* = 5773)	Treatment-related factors	3.3%	Venous
Zhou et al. (2010) [4]	ND lymphoma (*n* = 422)	Early treatment phase	64% of VTE in first 3 cycles	Venous
Hohaus et al. (2018) [12]	Lymphoma (*n* = 857)	Poor PS, bulky disease, high LDH, low albumin	PS associated with VTE risk (OR up to ~5.1)	LEDVT
Park et al. (2012) [13]	Lymphoma (*n* = 686)	Hypertension, LDH, CVC	9.1% vs. 5.3% (with vs. without CVC)	Venous
Byun et al. (2019) [14]	PCNSL (*n* = 235)	Low albumin, female gender	NR	Venous
Lim et al. (2016) [15]	DLBCL (*n* = 322)	Age, tumour burden, inflammatory status	NR	Venous
Zhang et al. (2016) [16]	Lymphoma with CVC (*n* = 565)	CVC	7.1% DVT	UEDVT
Lekovic et al. (2010) [7]	PMBCL (*n* = 42)	Mediastinal mass (compression)	35.7% (many at diagnosis)	Venous
Gebhart et al. (2014) [17]	Splenic MZL (*n* = 70)	LA (particularly post splenectomy)	NR	Venous
Yokoyama et al. (2012) [18]	DLBCL (*n* = 142)	Age, clinical factors	NR	Venous

BMI: body mass index; CNS, central nervous system; CVC, central venous catheter; DLBCL, diffuse large B-cell lymphoma; DVT, deep vein thrombosis; FL, follicular lymphoma; HL, Hodgkin lymphoma; LA, lupus anticoagulant; LEDVT, lower-extremity deep vein thrombosis; MZL, marginal zone lymphoma; ND, newly diagnosed; NHL, non-Hodgkin lymphoma; NR, not reported; PCNSL, primary central nervous system lymphoma; PMBCL, primary mediastinal B-cell lymphoma; PS, performance status; UEDVT, upper-extremity deep vein thrombosis; VTE, venous thromboembolism.

### 2.2. Lymphoma-Related Risk Factors

**Figure 1:**
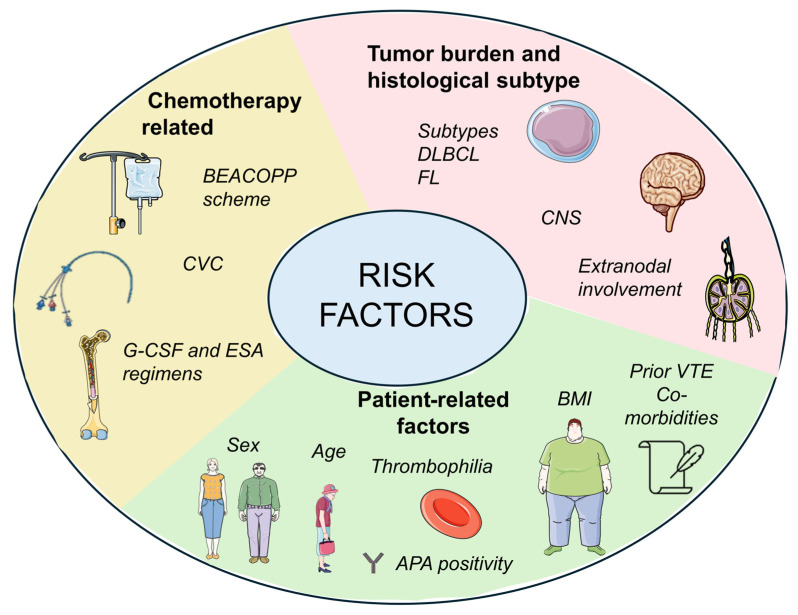
Risk factors for venous thromboembolism (VTE) in lymphoma.

In general, thrombosis, defined as blood clotting beyond that of physiological haemostasis, may be caused by three factors, collectively known as Virchow’s triad: vascular wall abnormalities, blood hypercoagulability, and reduced blood flow. In lymphomas, the pathogenesis of thrombosis is multifactorial, involving baseline patient characteristics, intrinsic tumour biology, host inflammatory responses and treatment-related influences, all of which promote thrombosis either by their complex interplay with factors pertaining to Virchow’s triad or directly by activating independent clotting pathways (Figure 1, Table 1).

#### 2.2.1. Histology

Regarding histologic subtypes, the majority of studies in the field report that aggressive lymphomas are associated with a greater risk of VTE than indolent types [5,6,9,11,19]. Diffuse large B-cell lymphoma (DLBCL), the most frequent subtype of the B-NHLs, shows a clearly higher IR for VTE than other, more indolent lymphoma categories, such as follicular lymphoma (FL) [10,12,20]. Numerically, the actuarial IR for DLBCL reaches 10–12% during the first year of diagnosis, whereas in low-grade lymphomas, the corresponding values vary between 1.5 and 4% [9,11]. Moreover, patients with DLBCL tend to develop more severe VTE episodes, based on the study published by Santi RM et al. [10]. T-non-Hodgkin lymphomas (T-NHL), a rather rare histologic subtype with generally poor prognosis, also fall under the high-risk category for VTE [12]. According to a comprehensive analysis of three prospective randomised trials by the German Hodgkin Study Group (GHSG), the VTE risk in patients with HL was 3.3%, with 193 VTE events in 5773 patients [2].

#### 2.2.2. Tumour Burden—Clinical Stage—Site of Disease

Initial disease staging parameters, such as tumour burden, clinical stage and involved anatomic sites, have been shown to affect VTE risk in lymphomas. More specifically, bulky disease and advanced stages according to the Ann Arbor classification system are associated with increased thrombotic risk. The meta-analysis of 29 cohort studies by Caruso et al. showed increased IR of VTE in patients with stage III (IR 6.9%, 95% Confidence interval (CI) 3.8–12.4) and stage IV disease (IR 11.5%, 95% CI 8.6–15.5) [5]. Advanced disease stage has also emerged in retrospective studies of NHL patients as a significant predictor of acute VTE in survival analysis models (HR 1.5; 95% CI, 1.2–1.7) [9]. Importantly, several experimental models support the theory that activation of coagulation pathways also stimulates tumour growth. In other words, thrombotic events reflect a more aggressive disease biology and, consequently, a worse prognosis [5,21]. Older studies demonstrated that disease localisation is another important risk factor for VTE, with PCNSL, mediastinal disease, and systemic lymphoma with CNS involvement conferring the highest risk [8,11].

#### 2.2.3. Laboratory Parameters

Complete blood count (CBC) parameters such as haemoglobin level, pre-chemotherapy platelet and white blood cell (WBC) count are included in the two most widely used RAMs for VTE in lymphomas, Khorana and Throly [22,23]. However, their effect on VTE occurrence remains uncertain. More specifically, according to the results of a network analysis of 21 studies published by Hohaus S et al., platelet count does not play a significant role as a VTE risk factor in lymphoma [24]. Regarding WBC count, data are controversial; leukocytosis, defined as a WBC > 11 × 10^9^/L has been reported to contribute to thrombotic risk in two studies, while neutropenia (<1 × 10^9^/L) during therapy is included in the Throly model as a risk factor based on the multivariate prognostic model analysis of the original study [15,20]. Similarly, findings regarding anaemia are inconsistent. Seven studies reported no association between pre-chemotherapy haemoglobin levels and VTE risk [2,12,15,20,25], and one study identified an association only in univariate analysis [9]. Only Antic et al. found anaemia (Hb < 10 g/L) to be a VTE risk factor in a multivariate setting [22].

Elevated LDH levels, which are associated with more aggressive disease biology, are also associated with thrombotic risk in several studies [11,12,13]. Furthermore, two studies, a monocentric retrospective analysis of 857 adults with newly diagnosed lymphoma from Europe and a multicenter study of 235 patients with newly diagnosed PCNSL in Korea, have shown a possible association between low albumin levels and the incidence of VTE [12,14]. Elevated beta_2_-microglobulin has also emerged as a potential risk factor for VTE; in a study of patients with chronic lymphocytic lymphoma (CLL), a beta_2_-microglobulin level above 4 mg/L was associated with increased thrombotic risk (HR 1.97; 95% CI: 1.14–3.41) [26].

#### 2.2.4. International Prognostic Index (IPI [19])

The International Prognostic Index (IPI) is a well-established and widely accepted prognostic tool for aggressive lymphomas. For the IPI calculation, several lymphoma- and patient-specific parameters are considered, such as clinical stage, LDH levels, number of extranodal sites, age and PS, most of which are associated with increased VTE risk, as discussed above [27]. Consequently, a high IPI score is associated with elevated thrombotic risk, a finding supported by several studies [3,15,25,28].

#### 2.2.5. Timing of Thrombotic Events

According to most studies, the cumulative incidence of VTE is highest in the first months following the initial diagnosis, with a significant decrease thereafter over time [10,14,15,25]. The higher tumour burden at diagnosis may explain this phenomenon. A prospective cohort study on 2720 patients (2037 DLBCL; 693 FL) by Sanfilippo et al. showed that in DLBCL the incidence of VTE per 1000 patient-years was 271 (95% CI: 203–363) during days 0–30, decreased to 108 (95% CI: 87–135) during days 31–180, and further declined to 35 (95% CI: 24–51) during days 181–365. Similarly, in patients with FL, the incidence of VTE per 1000 patient-years was 140 (95% CI: 70–280) during days 0–30, 55 (95% CI: 33–91) during days 31–180, and 23 (95% CI: 11–48) during days 181–365 [9]. In the same direction, the meta-analysis of 18 studies by Caruso et al. concluded that the majority (95%) of VTE events occurred during treatment, whereas only 1.2% occurred during the follow-up period [5]. Prior to chemotherapy initiation, the occurrence of VTE is particularly common among patients with local venous compression. Across two retrospective single-centre analyses, including 857 patients with all types of lymphoma and 500 DLBCL patients, respectively, the proportion of VTE events occurring before the start of therapy ranged from 16/80 (20%) to 54/95 (57%) among all observed VTE events in each study [12,28].

### 2.3. Individual Patient-Related Risk Factors and Host Inflammatory Responses

Patient-related risk factors for thrombosis have been extensively studied in cancer patients (CP), and these also broadly apply to patients with haematological malignancies. Below, we review studies that report patient-specific parameters associated with VTE risk, specifically in patients with lymphoma.

#### 2.3.1. Age

Advanced age has been identified as a risk factor for VTE in several studies assessing thrombosis in CP. In lymphoma, six retrospective studies have shown increased thrombotic risk in older patients with NHL, the majority of whom were diagnosed with DLBCL [10,12,14,15]. More specifically, in these studies, advanced age was defined as age 60 or older, and the odds ratio for VTE ranged from 1.6 to 3.3.

#### 2.3.2. Gender

Data on the role of gender as a VTE risk factor in lymphoma patients are conflicting. The majority of the studies assessed in the review by Hohaus et al. [12] showed no association, except for three studies in which female patients had a significantly higher probability of VT [4,10,14]. However, none of the aforementioned studies assessed the possible association between VTE IR and hormonal treatment, which is often prescribed in young female patients under chemotherapy either as contraception or for other individual medical reasons.

#### 2.3.3. Elevated Body Mass Index (BMI)

Obesity is an established independent risk factor for thrombosis beyond cancer and lymphoma patients, and as expected, it is included as a variable in lymphoma-specific risk assessment tools (ThroLy and Khorana). However, studies on the association between VTE and elevated body mass index (BMI) have yielded conflicting results. In the prospective cohort analysis of 2730 male patients with DLBCL (*n* = 2037) and FL (*n* = 693), BMI ≥ 30 kg/m^2^ was a significant risk factor for VTE in a competing risk model in the first year of diagnosis (adjusted HR 1.6; 95% CI 1.08–2.37) [9]. In another survey for long-term health outcomes on 2-year survivors following bone marrow transplantation (BMT) for NHL, BMI greater than 25 kg/m^2^ was associated with increased VTE risk [29].

#### 2.3.4. Performance Status

Limited mobility due to disease burden, hospitalisation or post-surgery is a strong predictor for patients’ poor PS, which further increases the underlying thrombotic risk. Most lymphoma-specific studies in the literature confirm this finding and report a negative association with reduced PS and VTE, with ORs ranging from 1.5 to 5 [12,15,20,22,25]. Mobility is the main determinant of PS; therefore, the role of PS as a thrombotic risk factor may vary widely across studies, depending on the clinical setting and histologic type. Regarding VTE localisation, in a monocentric retrospective analysis of 857 patients with lymphoma, Hohaus et al. found that poor PS was associated with a higher incidence of thrombosis in the lower extremities [12].

#### 2.3.5. Comorbidities

Comorbidities such as heart failure, renal or liver disease and chronic obstructive pulmonary disease have also been proposed as predictors for VTE in lymphoma patients. Moreover, the number of comorbidities poses a higher risk for thrombosis, with patients having three or more concurrent diseases, in addition to the lymphoma having a 4-fold higher probability of developing VTE [13]. Acute infections have also been associated with increased VTE risk based on the results of the retrospective analysis by Li et al. based on 325 NHL patients [30].

#### 2.3.6. History of VTE/Thrombophilia

A prior history of thrombosis is a well-established risk factor for recurrent events. However, this association has not been clearly demonstrated in patients with lymphoma, and the data are conflicting [9,20,31]. Most studies, however, have not assessed or reported these variables because prior thrombotic events are rare and thus unlikely to be risk factors for thrombosis. A history of previous VTE/MI/stroke is included as a predictive parameter in two of four published models (ThroLY and Tic-Lymho).

Antiphospholipid antibodies (APA) have been frequently reported in patients with malignancies, particularly lymphoid neoplasms [5]. However, the clinical significance of positive APA in lymphoma remains controversial [32,33]. In addition, there is a paucity of data regarding APA prevalence and clinically thrombotic potential among lymphoma patients using up-to-date diagnostic methods and diagnostic criteria. The contribution of hereditary and acquired thrombophilia to lymphoma-associated VTE has yet to be clearly defined. Likewise, the clinical relevance of lupus anticoagulant activity and deficiencies of antithrombin, protein C, or protein S in patients with lymphoma remains uncertain, owing to the heterogeneity of the available evidence [17,18] which further highlights the need for prospective evaluation.

### 2.4. Treatment-Related Risk Factors

The thrombotic potential of individual chemotherapeutic agents is difficult to assess, given the multi-agent regimens administered in most patients with lymphoma. However, most regimens are known to increase VTE risk. Higher-intensity and longer-duration regimens confer the highest risk. Regarding HL, the BEACOPP scheme for advanced stages is considered to have a higher thrombotic risk than ABVD, especially when administered every 14 days instead of 21 days (*p* = 0.0079) [2]. In a cohort of 2730 patients with NHL within the Veterans’ Administration Central Cancer Registry, doxorubicin-based treatment has emerged as an independent risk factor for thrombosis [9].

Furthermore, corticosteroids, particularly dexamethasone, have been implicated in cancer-associated thrombosis through endothelial activation, increased production of coagulation factors, and impaired fibrinolysis. Emerging evidence suggests that prolonged exposure and higher cumulative doses may further enhance thrombotic risk, while treatment with dexamethasone has been identified as a potential contributor to VTE in patients with lymphoma and multiple myeloma [34]. L-Asparaginase-containing regimens, which are mainly used in T-cell lymphoblastic lymphoma and acute B-cell lymphoblastic leukaemia/lymphoma, represent another well-established treatment-related thrombotic risk factor. Asparaginase induces depletion of natural anticoagulant proteins, particularly antithrombin, resulting in a marked procoagulant state. Venous thromboembolic events have been reported in approximately 14–23% of adult patients receiving asparaginase-based therapy, although rates may be even higher in selected populations (i.e., patients with CVC) [35,36].

Novel therapeutic approaches have also been associated with thrombotic complications. Immune checkpoint inhibitors (ICIs), such as Programmed Death-1 (PD-1) inhibitors, may promote thrombosis through the release of pro-inflammatory cytokines and activation of innate immunity, leading to systemic endothelial injury, platelet aggregation and inhibition of fibrinolysis. According to recently published data, mainly from studies on solid tumours, the pro-thrombotic state induced by ICIs may increase thrombotic risk in treated patients [37,38]. Likewise, CAR-T cell therapy has been associated with coagulation abnormalities and thrombotic complications, largely caused by immune system activation via cytokine release syndrome (CRS) and immune effector cell-associated neurotoxicity syndrome (ICANS). Both of these common CAR-T cell therapy complications lead to the release of several proinflammatory molecules, which in turn trigger complement activation and endothelial inflammation, particularly during the first days after infusion [39]. According to a recent review, the incidence of VTE among patients treated with CAR-T cell therapy ranges from 2% to 11%, with B-NHL patients having the highest rates of events [40].

Data regarding Bruton tyrosine kinase (BTK) inhibitors are more complex; although ibrutinib is primarily associated with bleeding due to platelet dysfunction secondary to inhibition of signalling pathways mediated by glycoprotein VI (GPVI), integrin αIIbβ3, and glycoprotein Ib (GPIb) [41], thrombotic events have been reported, suggesting a clear and substantial thrombotic role of BTK inhibitors. In a retrospective cohort of 565 patients with B-cell malignancies, including non-Hodgkin lymphomas, the incidence of venous and arterial thrombosis during ibrutinib treatment was low (1.7 events per 100 person-years). Nevertheless, the overall impact of BTK inhibitors on thromboembolic risk in lymphoma patients remains incompletely characterised and warrants further investigation [42]. Lenalidomide’s thrombotic risk has been extensively evaluated in studies for multiple myeloma. However, emerging data support its significant therapeutic potential in patients with lymphoma. The meta-analysis by Christos PJ et al. evaluated VTE in patients with B-NHL receiving lenalidomide and showed that thrombotic risk is similar to that in multiple myeloma and lower with lenalidomide combined with a biologic agent (0.49/100 patient-cycles) compared with single-agent lenalidomide (1.07) or its combination with chemotherapy (0.89).

The thrombogenic potential of lenalidomide has been extensively evaluated and reported in patients with multiple myeloma. The meta-analysis by Yamshon et al. assessed VTE in patients with B-NHL treated with lenalidomide and showed that the thrombotic risk is similar to that in multiple myeloma and lower with lenalidomide combined with a biologic (0.49/100 patient-cycles) than with single-agent lenalidomide (1.07) or its combination with chemotherapy (0.89) [43].

Indwelling CVC are commonly used in patients with lymphoma and are associated with increased risk of thrombosis [11,13]. There are few studies in the literature addressing specifically the VTE risk of CVC in lymphoma patients. In the Asian cohort study by Zhang X et al. on 565 patients with B-NHL, T-NHL and HL, the rate of upper-extremity deep vein thrombosis (UEDVT) associated with the presence of CVC was 7.1% (40/565) without significant differences between lymphoma subtypes; however, the incidence was significantly higher compared to patients with other malignancies (209/7463; 2.80%, *p* < 0.001) [16].

Granulocyte colony-stimulating factor (G-CSF) and erythropoiesis-stimulating agents (ESA) are supportive regimens commonly used in lymphoma patients during treatment to mitigate chemotherapy-related myelotoxicity. Data regarding cancer patients support their increased thrombotic risk. However, there are no sufficient data addressing their association with VTE in patients with lymphoma [23].

## 3. Emerging Biomarkers for Venous Thromboembolic Risk

**Figure 2:**
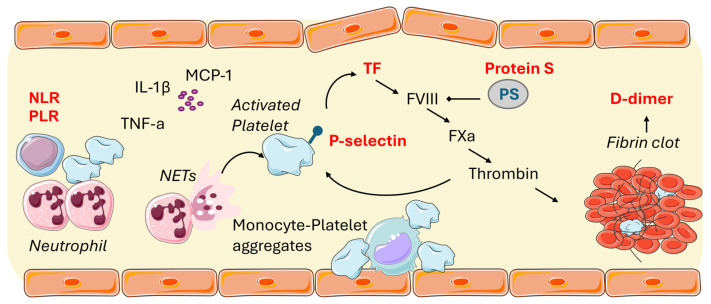
Biomarkers associated with thrombosis risk in lymphoma.

Table 2 and Figure 2 summarise data on biomarkers for VTE in lymphoma patients. The development of biomarkers for VTE has historically focused on downstream effector mechanisms rather than upstream triggers of thrombosis [44]. Among these biomarkers, D-dimer remains one of the oldest and most widely used indicators of thrombotic activity. Fibrin degradation products were first characterised in the 1960s, followed by the identification of D-dimer in 1973 [45]. Despite initial limitations in measuring D-dimer, it has become a central biomarker for diagnosing and monitoring VTE.

Higher D-dimer levels are associated with increased risk of VTE in lymphoma patients, and are frequently incorporated into risk models and machine learning algorithms for VTE prediction [34,46,47,48]. Several studies have highlighted the predictive role of D-dimer levels. In a cohort of 157 newly diagnosed HL and NHL patients, elevated D-dimer levels (>500 ng/mL) and pre-existing cardiovascular comorbidities were identified as independent predictors of VTE in multivariate analysis. Patients with cardiovascular disease had a significantly higher incidence of VTE compared with those without comorbidities, and elevated D-dimer levels were strongly associated with increased thrombotic risk (15.8% vs. 1.2%). In contrast, other clinical variables—including sex, age, BMI, disease stage, extranodal involvement, histological subtype, and treatment regimen—were not significantly associated with VTE risk in the multivariate model [49].

P-selectin is an adhesion molecule belonging to the selectin family that is expressed by several cell types, including platelets, endothelial cells, leukocytes, and certain tumour cells. Because activated platelets exhibit high levels of P-selectin on their surface, this molecule plays a central role in the pathogenesis of thrombosis, particularly in CAT and haematologic malignancies such as lymphoma. Through its interaction with P-selectin glycoprotein ligand-1 (PSGL-1) on leukocytes, P-selectin promotes platelet–leukocyte aggregation, endothelial activation, and tissue factor expression, thereby amplifying coagulation and thrombus formation. Importantly, soluble P-selectin (sP-selectin), released into the circulation following platelet and endothelial activation, can be readily measured in plasma and has emerged as a clinically accessible biomarker. Elevated sP-selectin levels have been associated with an increased risk of VTE and may also reflect tumour burden and disease activity [48,50]. Evidence supporting the prognostic value of P-selectin was reported. In the Vienna Cancer and Thrombosis Study (CATS), which included patients with haematologic malignancies, median P-selectin levels were significantly higher in patients who developed VTE than in those who did not [51]. Patients with sP-selectin levels at or above the 75th percentile had a significantly increased risk of VTE, with a hazard ratio of 2.6 (95% CI 1.4–4.9).

Inflammatory biomarkers are altered in patients with lymphoma who develop VTE. Significantly higher levels of neutrophil-to-lymphocyte ratio (NLR), platelet-to-lymphocyte ratio (PLR), erythrocyte sedimentation rate (ESR), C-reactive protein (CRP), and lactate dehydrogenase (LDH), as well as lower total protein and albumin levels [52], have been reported in patients with lymphoma and VTE compared to lymphoma patients with no VTE. In multivariate analysis, NLR and CRP emerged as independent predictors of VTE events (*p*  =  0.046, OR  =  1.043, 95% CI: 1.001–1.087 and *p*  =  0.024, OR  =  1.007, 95% CI: 1.001–1.013, respectively). ROC analysis demonstrated acceptable predictive performance for NLR, PLR, and CRP [52] (Sensitivity (Sn)  =  65.2%, Specificity (Sp)  =  57.1% for NLR; Sn  =  69.6%, Sp  =  49.7% for PLR; and Sn  =  71.7%, Sp  =  63.7% for CRP) (*p* = 0.001 for all). These findings support the concept that systemic inflammation contributes to the development of VTE in lymphoma and suggest that readily available inflammatory markers may aid in thrombotic risk stratification. TNF-α and P-selectin were elevated in lymphomas, particularly in DLBCL with thrombosis, whereas IL-1β, tissue factor (TF), and P-selectin were markedly increased in aggressive DLBCL and DLBCL-like lymphomas. In FL, monocyte chemoattractant protein (MCP-1) and TGF-β were reduced, whereas thrombin levels were increased. Overall, IL-1β, P-selectin, TF, and fibrinogen were elevated in FL and HL compared with healthy controls, indicating enhanced inflammatory and procoagulant activity. Neutrophils are continuously recruited during chronic inflammation and contribute by forming neutrophil extracellular traps (NETs). In clinical and translational studies, NETs are typically assessed indirectly through circulating biomarkers such as cell-free DNA, citrullinated histone H3 (Cit-H3), myeloperoxidase (MPO)-DNA complexes, and neutrophil elastase-DNA complexes, rather than by direct quantification of NET structures or the activation of other immune cells. Specifically, NETs have been implicated in thrombus formation through their capacity to trap platelets, promote TF activity, and amplify coagulation [53]. NETs are elevated in HL and correlate with thrombotic risk [48]. Inflammatory cytokines and tissue factor-carrying monocytes are mechanistically linked to thrombosis in lymphoma, but their direct predictive value is still under investigation [48]. These findings suggest that inflammatory dysregulation contributes to thrombotic risk in lymphoma and that simple, widely available inflammatory markers may help identify patients at increased risk of VTE.

Elevated factor VIII activity has been consistently associated with an increased risk of incident VTE in lymphoma cohorts, reflecting the hypercoagulable state frequently observed in malignancy [34]. In addition, reduced total protein S activity, an important natural anticoagulant, has been associated with increased thrombotic risk, suggesting that impaired anticoagulant pathways may contribute to thrombogenesis in these patients. Platelet activation also appears to play a significant role; increased levels of platelet–monocyte aggregates and platelet-associated tissue factor have been reported in certain lymphoma subtypes and are associated with thrombotic events, highlighting the interplay between inflammation, cellular activation, and coagulation.

Genetic predisposition may further modulate thrombotic risk in lymphoma. Variants such as Factor V Leiden, serpinA10 rs2232698, and factor XIII-A Val34Leu have been associated with increased susceptibility to VTE, although their clinical utility for routine risk stratification remains under investigation [34,50]. Additional haemostatic abnormalities, including impaired fibrinolysis [54], may contribute to the persistence of thrombi once formed. Other laboratory markers—such as elevated fibrinogen levels, enhanced thrombin generation, and increased mean platelet volume—have shown some predictive value for thrombotic complications; however, findings across studies remain inconsistent, and further research is needed to determine their role in clinical RAMs for lymphoma-associated thrombosis. P-selectin mediates platelet–leukocyte adhesion, microparticle generation and thrombin formation and reflects platelet and endothelial activation. In cancer patients high soluble p-selectin levels are associated with a 2–3 times higher risk of VTE [55]. D-dimer levels are known to increase following thrombosis, but elevated d-dimer is also a strong predictor of future thrombotic risk in cancer patients, independent of tumour type and stage [56]. Both d-dimer and p-selectin levels have been incorporated into RAMs, adding incremental predictive value [57,58].

**Table 2:** Biomarkers associated with venous thromboembolism in Lymphoma patients.

Biomarker	Study/Reference	Population	Main Findings	Association with VTE
D-dimer	Nguyen et al. [49]	57 newly diagnosed HL/NHL patients	D-dimer > 500 ng/mL predicted VTE; VTE incidence 15.8% vs. 1.2% with normal levels	Independent predictor
Soluble P-selectin (sP-selectin)	Ay et al. (Vienna CATS) [55,59]	Cancer patients including haematologic malignancies	Median sP-selectin significantly higher in patients with VTE; sP-selectin ≥ 75th percentile associated with increased VTE risk	HR 2.6 (95% CI 1.4–4.9)
Neutrophil-to-Lymphocyte Ratio (NLR)	Otasevic et al. [52]	Lymphoma	Higher NLR in patients with VTE; independent predictor in MVA	OR 1.043 (95% CI 1.001–1.087)
C-reactive Protein (CRP)	Otasevic et al. [52]	Lymphoma	Elevated CRP independently associated with VTE	OR 1.007 (95% CI 1.001–1.013)
Platelet-to-Lymphocyte Ratio (PLR)	Otasevic et al. [52]	Lymphoma	Elevated in VTE patients; predictive performance in ROC analysis	Significant in ROC analysis, not independent predictor
ESR	Otasevic et al. [52]	Lymphoma	Higher in patients developing VTE	Associated in univariate analysis
LDH	Otasevic et al. [52]	Lymphoma	Higher in VTE group; reflects aggressive disease biology	Associated with VTE risk
Albumin	Hohaus et al. [24]; Byun et al. [14]	Newly diagnosed lymphoma; PCNSL	Low albumin associated with increased VTE incidence	Potential predictor
Factor VIII activity	Multiple studies summarised by Heit et al. [60]	Lymphoma	Elevated levels consistently associated with hypercoagulability and incident VTE	Strong biological association
Protein S activity	Heit et al. [60]	Lymphoma	Reduced activity associated with increased thrombotic risk	Potential predictor
Platelet–monocyte aggregates/Platelet-associated tissue factor	Heit et al. [60]	Selected lymphoma subtypes	Increased platelet activation associated with thrombosis	Mechanistic association
NETs (cfDNA, MPO-DNA, Cit-H3)	Zivkovic et al. [48]	Lymphoma	NET formation elevated in lymphoma and linked to thrombotic risk	Emerging biomarker
Tissue Factor (TF)	Zivkovic et al. [48]	DLBCL and aggressive lymphomas	Increased TF expression in aggressive lymphoma and thrombosis	Emerging biomarker
TNF-α	Zivkovic et al. [48]	DLBCL	Elevated in lymphoma patients with thrombosis	Emerging biomarker
IL-1β	Zivkovic et al. [48]	Aggressive DLBCL, FL, HL	Elevated compared with healthy controls; associated with procoagulant state	Emerging biomarker
Fibrinogen	Zivkovic et al. [48]	FL and HL	Increased levels compared with controls	Emerging biomarker

Based on available data, D-dimer, factor VIII, protein S activity, and P-selectin have emerged as the most promising biomarkers for thrombotic risk in lymphoma. D-dimer, and factor VIII to a lesser extent, stand out as the most clinically accessible. Protein S activity and P-selectin have questionable clinical utility at the moment, given their limited availability in many clinical settings and the lack of robust standardisation. Genetic markers and thromboinflammatory parameters represent emerging areas of research.

Multiple haemostatic and inflammatory biomarkers have been associated with increased risk of VTE in lymphoma patients. Tissue factor (TF) binds to activated factor VII (VIIa), forming a complex (TF/VIIa) that initiates coagulation cascade by activating factor IX and X, with consequent thrombin generation and fibrin formation. Thrombin, in turn, triggers platelet activation with subsequent release of platelet granule content and amplification of the whole activatory process. D-dimer and soluble P-selectin are the most consistently validated markers, reflecting activation of coagulation and platelet–endothelial interactions, respectively. Additional contributors include inflammatory markers (e.g., NLR, PLR, cytokines), neutrophil extracellular traps (NETs), and procoagulant factors such as tissue factor and elevated factor VIII activity, highlighting the link between inflammation and hypercoagulability. Reduced protein S activity, platelet–monocyte aggregates, impaired fibrinolysis, and prothrombotic genetic variants—may further modulate thrombotic risk, although their clinical predictive value remains less well established. CRP, C-reactive protein; IL-1β, interleukin-1 beta; NETs, neutrophil extracellular traps; NLR, neutrophil-to-lymphocyte ratio; PLR, platelet-to-lymphocyte ratio; TF, tissue factor; TNF-α, tumour necrosis factor alpha; and VTE, venous thromboembolism.

## 4. Risk Assessment Models for VTE Risk

International guidelines recommend systematic assessment of VTE risk in all patients with malignancy [61]. There are several RAMs for cancer patients, which are therefore not lymphoma-specific. These are summarised in Table 3a. Historically, the most widely used cancer risk-assessment tool is the Khorana score, originally developed for ambulatory patients receiving chemotherapy, which assigns one point to lymphoma as a high-risk tumour sit [23]. Although lymphoma represented 12.1% of the derivation cohort, subsequent evaluations in lymphoma populations have yielded inconsistent results [2,3,62,63]. A meta-analysis by van Es et al. has shown that the predictive performance of the Khorana score varies across cancer types and that its sensitivity is limited [64] since the majority of VTE events occur outside the high-risk group. These findings underscore that the pan-cancer Khorana model does not adequately capture lymphoma-specific risk features and should be applied with caution in this setting. The Vienna CATS Score [55] expands the Khorana Risk Score by incorporating D-dimer and soluble P-selectin, improving its predictive performance, with a positive predictive value of 22.1%, a negative predictive value of 94.9%, a sensitivity of 31.9%, and a specificity of 91.9%, but limiting its generalizability given the limitations of P-selectin assessment in routine clinical practice. In the Cancer and Thrombosis Study (CATS), tumour-site category was the only Khorana parameter independently associated with VTE; combining tumour-site classification with D-dimer levels yielded a simplified model validated in an independent cohort, although lymphoma patients were underrepresented in the validation set [65]. In the more recent Electronic Health Record (EHR) CAT, lymphoma patients represented 9.6% of the derivation and 7.7% of the validation cohort [66]. High-grade NHL emerged as a high-risk characteristic compared to low-grade NHL and HL and the model included 11 parameters in total, assigning patients as low or high risk.

RAMs that have included only lymphoma patients are presented in Table 3b. ThroLy is a lymphoma-specific risk model developed in a large cohort of 1820 patients, including aggressive and indolent NHL and HL. It is a 7-parameter score based on clinical and laboratory patient data, including previous VTE, reduced mobility, obesity, extranodal disease, mediastinal involvement, haemoglobin < 10 g/dL, and platelet count > 350 × 10^9^/L. Patients are stratified into low-, intermediate-, or high-risk categories for VTE based on their cumulative score. In internal validation cohorts, ThroLy showed high discrimination and adequate risk stratification of patients according to their thrombotic risk [67]. However, external evaluation of 428 patients with DLBCL has demonstrated substantially lower performance, with nearly half of the thrombotic events occurring in patients classified as low risk [20]. Similar limitations were observed in another single-centre cohort study of 208 patients (90% NHL, 10% HL), highlighting challenges to generalizability [22].

Dharmavaram et al. developed a model based on a cohort study of 790 patients with DLBCL or FL. The variables included in the multivariate model are lymphoma subtype (FL versus DLBCL) (aHR 0.36, *p* = 0.10), albumin (per unit increase) (aHR 0.47, *p* = 0.01), leukocytosis greater than 11,000 (aHR 1.71, *p* = 0.30), and bulky disease (lesion greater than 10 cm) (aHR 1.82, *p* = 0.23). High-risk patients had significantly increased VTE risk, with a 2-year c-statistic of 0.79 [63]. The TiC-LYMPHO score integrates the presence of a mediastinal mass, Ann Arbor stage, four genetic risk variants (rs6025, rs4524, 3 rs5985, and SERPINA10 rs2232698), ≥3 days immobilisation, lymphoma subtype, and prior VTE, achieving a derivation c-statistic of 0.78 [62]. However, both models lack external validation. In addition, the TiC-LYMPHO score is not easily applicable in everyday clinical practice.

Hohaus et al. developed a simplified three-parameter score based on CNS involvement, bulky disease, and PS. CNS involvement defines the highest-risk group; patients with bulky disease and/or impaired PS are classified as high risk, and patients without any of these parameters are considered standard risk for thrombosis [12]. When compared with Khorana and ThroLy, this model demonstrated adequate performance, identifying 82% of VTE events in a high-risk group, with confirmatory independent validation cohorts still pending.

A retrospective cohort study in lymphoma patients from the Veterans Affairs system was used to develop a Fine and Gray subdistribution hazard model for predicting VTE and PE/LE-DVT, with external validation in cohorts from Harris Health System and MD Anderson [68]. The model incorporated variables such as lymphoma histology, BMI, treatment type, recent hospitalisation, prior VTE, immobilisation, and time to treatment, achieving c-statistics of 0.68 in the derivation cohort and 0.69–0.72 in the validation cohorts, providing a simple, clinically based model for personalised VTE risk prediction in lymphoma patients. A retrospective study in China involving 790 patients with newly diagnosed lymphoma used LASSO selection and multivariable Cox modelling to develop a nomogram for VTE risk, with internal validation. Predictors included ECOG performance status, prior VTE, coronary artery disease, CVC use, and APTT. The model had an AUC 0.81 and 0.73 in validation and outperformed conventional scores such as ThroLy and Khorana, with good calibration [69].

Models using machine learning algorithms (e.g., gradient boosting machines, random forest) have achieved high predictive accuracy (AUC up to 0.95) by integrating a broader set of clinical and laboratory features, including D-dimer, LDH, CVC, and ECOG status. These models facilitate early identification of high-risk patients and may guide individualised prophylaxis in the future [47,70].

Overall, existing evidence highlights the lack of a “perfect” risk-stratification model and the need for robust, externally validated, lymphoma-specific risk-prognostic tools. Because VTE risk may evolve during treatment—due to immobilisation, CVC placement, or changes in therapy—future efforts should also consider dynamic prediction models to better guide individualised thromboprophylaxis strategies. Table 3 summarise the main lymphoma-specific risk assessment models.

**Table 3:** (**a**) VTE risk assessment models in cancer patients (including lymphoma); (**b**) VTE risk assessment models in lymphoma patients.

Model	Population	Risk Factors/Variables	Risk Groups Performance	c-Statistic
(**a**)				
Khorana Score (2008) [23]	Ambulatory cancer patients on chemotherapy *n* = 2701 (12.1% lymphoma)	Cancer type, PLT ≥ 350 × 10^9^/L, Hb < 10 g/dL, WBC > 11 × 10^9^/L, BMI ≥ 35 kg/m^2^	Variable performance in lymphoma; many VTE events outside high-risk group	0.7
EHR-CAT Li (2023) [66]	Derivation cohort (*n* = 9769, 9.6% lymphoma) Validation cohort (*n* = 79,517, 7.7% lymphoma)	Risk factors in final model: Cancer subtype, Stage III–IV disease, chemotherapy, all Khorana variables, prior VTE, immobility, recent hospitalisation > 3 days in previous 3 months, Asian/Pacific Islander race (protective)	High (≥3 points) vs. low (0–2 points) risk group; not assessed in lymphoma High-grade NHL assigned 2 points vs. HL and low-grade NHL 0 points	0.71 and 0.68
Meta-analysis van Es (2020) [64]	Mixed cancer population *n* = 3292 Lymphoma number NR	Khorana variables	Limited sensitivity; many events outside high-risk group	NR
ONKOTEV Cella (2017) [71]	Mixed cancer population *n* = 843 Lymphoma number NR	Khorana + metastatic disease, vascular compression, prior VTE	Improved prediction vs. Khorana; not lymphoma-specific	NR
TiC-Onco-Muñoz Martin (2018) [72]	Mixed cancer population *n* = 391 Lymphoma not included	Clinical (BMI > 25, Primary tumour site, Stage) + genetic variables	Improved predictive accuracy; limited lymphoma representation	NR
CATS model = Pabinger (2018) [65]	Cancer patients *n* = 1423 (Lymphoma 17.5%)	Tumour site + D-dimer	Improved prediction; lymphoma underrepresented	NR
(**b**)				
Khorana validation Santi (2017) [10]	NHL *n* = 1717 [pooled 12 clinical trials of Fondazione Italiana Linfomi (FIL)]	Khorana variables	Higher score associated with increased 6-month VTE	NR
Khorana validation Rupa-Matysek (2017) [3]	Lymphoma *n* = 428 (DLBCL = 241, HL = 187)	Khorana variables	Poor discrimination	0.51 (Overall population)
Khorana in lymphoma Dharmavaram (2020) [63]	*n* = 790 DLBCL + FL	Khorana variables	Score ≥ 3 linked to higher 2-year VTE risk	NR
ThroLy Antic (2016) [22]	Lymphoma (*n* = 1820)	Prior VTE, obesity, mediastinal involvement + others	Good internal discrimination; effective stratification	NR (reported as “high”)
ThroLy validation Rupa-Matysek (2018) [20]	*n* = 428 (DLBCL = 241 HL = 187)	ThroLy variables	Reduced performance; ~50% VTE in low-risk group	0.55
ThroLy validation Abdel-Razeq (2021) [67]	*n* = 524, all DLBCL	ThroLy variables	Adequate discrimination in some cohorts	NR
Lymphoma model Dharmavaram (2020) [63]	*n* = 790 (DLBCL = 542 FL = 248)	Lymphoma subtype, albumin, WBC > 11 × 10^9^/L, bulky disease (>10 cm)	High-risk group increased VTE risk	0.79
TiC-LYMPHO Bastos-Oreiro (2021) [62]	Lymphoma *n* = 208	Lymphoma subtype, mediastinal involvement, stage, bed rest > 3 days, FH or PH of VTE + genetic variables as per TiC-ONCO	Good derivation performance; no external validation	0.78
Modified ThroLy Li (2024) [30]	NHL *n* = 325	Adjusted Hb (<11 g/L), D-dimer (≥1345 ng/mL) + TAT + TM	Improved prediction; high PPV/NPV	NR
Lymph-CAT Ma (2024) [68]	Lymphoma *n* = 13,025	Histology, BMI ≥ 35, therapy, hospitalisation > 3 days, VTE history, immobilisation, time to treatment	Improved discrimination; clear risk group separation	0.68
ML VTE model He (2025) [47]	Hospitalised lymphoma patients (*n* = 605)	Multidomain variables: anticoagulant use, D-dimer, LDH, CVC, CEA, ECOG, total protein, cholesterol, infection (top predictors)	Excellent discrimination; identifies high-risk patients for early intervention; VTE incidence 10.1%	AUC = 0.953–0.954

AUC, area under the curve; BMI, body mass index; CVC, central venous catheter; DLBCL, diffuse large B-cell lymphoma; FH, family history; FL, follicular lymphoma; Hb, haemoglobin; HL, Hodgkin lymphoma; NHL, non-Hodgkin lymphoma; NR, not reported; NPV, negative predictive value; PH, personal history; PLT, platelet count; PPV, positive predictive value; TAT, thrombin–antithrombin complex; TM, thrombomodulin; VTE, venous thromboembolism; WBC, white blood cell count.

## 5. VTE Treatment and Thromboprophylaxis

From a clinical perspective, we need optimal VTE risk stratification in order to guide the use of thromboprophylaxis. Despite the well-documented thrombotic burden in these patients, there are currently no universally accepted lymphoma-specific guidelines for treatment or primary thromboprophylaxis. Clinical practice currently relies largely on recommendations derived from broader CAT guidelines, which do not fully capture the unique biological and clinical features of lymphoma.

### 5.1. Thromboprophylaxis

International guidelines from organisations such as the American Society of Haematology [73], the International Society of Thrombosis and Haemostasis (ISTH) [74] and the European Society for Medical Oncology (ESMO) [75] consistently recommend against routine thromboprophylaxis for all cancer patients receiving therapy. Instead, prophylaxis is reserved for selected high-risk individuals, reflecting the need to balance thrombotic risk against bleeding complications and treatment burden. This cautious approach is particularly relevant in lymphoma, where disease heterogeneity and variable risk profiles complicate the development of uniform recommendations.

Hospitalisation and reduced mobility—often assessed using performance status scales such as ECOG—is a consistent and significant risk factor for VTE and has been identified in the majority of lymphoma-focused studies. Thromboprophylaxis in this patient group is more clearly defined, where prophylactic administration of LMWH or fondaparinux in patients with adequate renal function is recommended [76].

Thromboprophylaxis is more consistently recommended for hospitalised patients with active malignancy in the absence of contraindications. The application of these recommendations to lymphoma remains challenging. The NCCN guidelines recommend using the Khorana score to guide risk stratification and thromboprophylaxis, and suggest primary prophylaxis in patients receiving systemic antineoplastic treatment who are at intermediate-to-high risk of thrombosis, as identified by malignancy type or a validated risk model (i.e., Khorana score ≥ 2). However, as discussed previously, pan-cancer risk models demonstrate limited predictive performance in lymphoma, and lymphoma-specific models, such as the ThroLy score, have shown inconsistent performance across external validation studies. Consequently, no risk assessment model has yet been universally adopted to guide decisions on thromboprophylaxis in lymphoma.

Regarding the appropriate agent for prophylaxis, in randomised controlled trials and prospective cohort studies that evaluated LMWH, haematologic malignancies accounted for 8.4% to 10.4% of the included cancer types, and results for the lymphoma subgroup were not reported. Data published on the efficacy and safety of anticoagulation in patients with lymphoma are scarce. In the randomised controlled trials comparing the use of LMWH with factor Xa inhibitors (apixaban, edoxaban, rivaroxaban) for treatment [77,78,79,80], patients with lymphoma accounted for approximately 5% to 10%, and no subgroup data were reported.

Available evidence and guidelines support the use of LMWH, apixaban, or rivaroxaban for thromboprophylaxis in cancer patients [75,81,82] and the recommendation extends to lymphoma patients. To date, there are no head-to-head comparisons of different oral anticoagulants for the prophylaxis of CAT, and indirect comparisons between trials are problematic. Evidence supporting primary thromboprophylaxis with direct oral anticoagulants in ambulatory cancer patients derives primarily from the AVERT and CASSINI trials. In the AVERT trial [83], 574 ambulatory patients with cancer initiating systemic therapy and a Khorana score ≥ 2 were randomised to apixaban 2.5 mg twice daily or placebo. Apixaban significantly reduced the incidence of VTE (4.2% vs. 10.2%), although at the cost of an increased risk of major bleeding (3.5% vs. 1.8%). Importantly, lymphoma was well represented, accounting for approximately 25% of the study population (145 patients). In the CASSINI [84] trial, 841 high-risk ambulatory cancer patients (7% lymphoma) were randomised to rivaroxaban 10 mg daily or placebo. Although the primary endpoint did not reach statistical significance in the intention-to-treat analysis (6.0% vs. 8.8%), rivaroxaban significantly reduced thromboembolic events during the treatment period, with low rates of major bleeding (2.0% vs. 1.0%). Current recommendations for thromboprophylaxis in lymphoma are largely extrapolated from these mixed-cancer populations rather than derived from dedicated lymphoma-specific randomised trials.

Novel therapies have further increased the complexity of managing lymphoma patients, and drug–drug interactions and drug-specific side-effects need to be considered when deciding who is the correct candidate for thromboprophylaxis and which agent should be prescribed.

Drug–drug interactions (particularly with CYP3A4 substrates), drug availability, haematological parameters, lymphoma localisation and patient preference should also be taken into consideration. Gastrointestinal involvement may interfere with the absorption of oral anticoagulants. In addition, all trials assessing oral anticoagulants excluded patients with a platelet count below 50 × 10^3^ or a very high risk of bleeding. Individualised decisions should also take into account aggressive histologic subtypes, advanced-stage disease, high tumour burden, impaired PS, recent hospitalisation, CVC placement, and early treatment phase, during which the risk of thrombosis is highest. Other factors, such as comorbidities and prior VTE, should also be incorporated into decision-making. Emerging biomarkers and more refined risk models may help identify patients most likely to benefit from prophylaxis in the future; however, their integration into clinical decision-making requires prospective validation.

Overall, current evidence supports a risk-adapted and individualised approach to thromboprophylaxis in lymphoma rather than a universal strategy. We require studies to define lymphoma-specific thresholds for intervention and to evaluate the clinical benefit of prophylaxis in biomarker-driven or dynamically stratified patient populations.

### 5.2. Treatment

There are no specific guidelines for VTE management in patients with lymphoma. Consequently, treatment strategies are extrapolated from international recommendations for CAT (ISTH and American Society of Clinical Oncology guidelines) [61,85]. Current guidance supports the initial administration of LMWH, UFH, fondaparinux, and direct factor Xa inhibitors as first-line therapy for VTE in patients with adequate renal function (defined as a creatinine clearance ≥ 30 mL/min). Current international guidelines recommend therapeutic anticoagulation for 3–6 months following CAT [81,86]. Beyond the acute phase, decisions regarding long-term anticoagulation require an individualised approach, taking into account thrombotic and bleeding risks, cancer activity, metastatic disease, treatment tolerability, life expectancy and patient preferences. Observational data suggest that the risk of recurrent VTE may persist even after six months, indicating the need for continued vigilance and individualised decision-making [87]. There are no lymphoma-specific guidelines regarding the optimal duration of anticoagulation after a thrombotic event. Disease status plays a crucial role in determining both the aetiology of VTE and the appropriate duration of anticoagulation. Disease remission justifies a shorter course of anticoagulation, particularly taking into account that VTE risk is higher during the first months following diagnosis. In patients with active, symptomatic disease or ongoing therapy, however, anticoagulation may continue beyond 6 months [88]. It should be noted that the evidence guiding anticoagulation beyond 6 months remains limited, and most recommendations are based on expert consensus rather than randomised trials. Recently, the API-CAT trial provided important data for extended treatment, demonstrating that reduced-dose apixaban (2.5 mg twice daily) was non-inferior to full-dose apixaban (5 mg twice daily) for the prevention of recurrent VTE in patients with active cancer who had completed at least 6 months of anticoagulation, while resulting in fewer clinically relevant bleeding events [89].

Special clinical scenarios further complicate management. In cases of catheter-related thrombosis, anticoagulation is recommended for at least 3 months and should be continued as long as the CVC remains in place [85]. However, there is no standardised approach or official guidance on the optimal duration of anticoagulation. Additionally, thrombocytopenia, which is frequently observed in patients receiving chemotherapy, poses challenges for safe anticoagulation. Dose adjustments of anticoagulants are generally advised when platelet counts fall below 50 × 10^9^/L [61,85,90]. Below this cut-off, decisions on treatment necessity and dosage require careful evaluation of bleeding risk.

Direct oral anticoagulants, as already discussed in the thromboprophylaxis setting, have emerged as a promising alternative to LMWH in the treatment of CAT. Evidence from several RCTs demonstrates that direct factor Xa inhibitors are noninferior to LMWH in the management of VTE [78,79,80,91]. However, these studies included only a small proportion of patients with haematologic malignancies, and lymphoma patients represented less than 10% of the study population. In fact, the data for all anticoagulants, parenteral and oral, are generally limited and require cautious interpretation, particularly with regard to specific cancer subgroups.

## 6. Discussion

This review highlights the complex, multifactorial nature of VTE in lymphoma, emphasising that thrombotic risk in this population is driven by a dynamic interplay among tumour biology, host-related factors, treatment exposures, and systemic inflammation. Lymphoma represents a distinct clinical entity within CAT, for which available predictive tools and management strategies remain suboptimal.

The multiparametric VTE risk in these patients is also time-dependent and evolving throughout the disease course. Importantly, the relative contribution of individual variables remains inconsistent across studies, reflecting heterogeneity in patient populations and highlighting the limitations of current risk-stratification approaches. Widely used pan-cancer risk models perform poorly in lymphoma patients. The Khorana score remains the most commonly applied tool in clinical practice, but its predictive accuracy is modest, and a substantial proportion of thrombotic events occur outside high-risk categories. Lymphoma-specific models, such as the ThroLy score, represent an important step toward tailored risk assessment; however, their external validation has significant limitations, including reduced discriminative performance and poor generalisability across cohorts. Given the heterogeneity of lymphoma subtypes, there is the risk that a uniform RAM for VTE in lymphoma may not fit all satisfactorily. To retain discriminative power, such a RAM would have to account for distinct lymphoma subtypes. In addition, most RAMs assign patients to more than two risk categories; a categorisation into either high or low risk for VTE would, however, facilitate decisions on thromboprophylaxis.

In this context, emerging biomarkers represent a promising avenue for improving VTE risk prediction, but the translation of biomarker data into clinical practice remains limited. Most biomarkers lack standardisation, prospective validation, and clear thresholds for clinical decision-making. Similarly, novel predictive models—including those incorporating machine learning—demonstrate improved performance but are not yet widely applicable due to complexity, lack of external validation, and limited accessibility.

The absence of lymphoma-specific guidelines for thromboprophylaxis represents a major unmet need. Current recommendations largely extrapolate from studies in solid tumours and advocate a risk-adapted approach using LMWH, apixaban or rivaroxaban for selected high-risk patients. However, given the limitations of existing risk models, identifying patients who would derive the greatest benefit from prophylaxis remains challenging. Moreover, the increasing complexity of lymphoma therapies, including novel targeted agents, introduces additional considerations such as drug–drug interactions and bleeding risk, further complicating clinical decision-making.

Future research efforts should prioritise the validation and refinement of existing lymphoma-specific risk assessment tools. In particular, prospective, multicentre validation studies are needed to confirm the clinical utility of lymphoma-specific scores such as Throly across diverse patient populations and treatment settings. In parallel, incorporating additional biomarkers—including D-dimer, P-selectin, inflammatory indices, and thrombin generation markers—into existing models may enhance predictive accuracy and enable more robust risk stratification. Equally important is the shift toward personalised medicine approaches. The integration of genetic and molecular profiling, including prothrombotic genetic variants and tumour-specific biological features, could refine individual risk prediction and facilitate tailored thromboprophylaxis strategies.

Moreover, future studies should explore dynamic risk assessment models that account for temporal changes in thrombotic risk over the disease course, particularly across treatment phases, hospitalisation, and evolving clinical status. The application of machine learning and artificial intelligence to large, real-world datasets may further advance the development of adaptive, clinically applicable prediction tools. Finally, there is a need for randomised clinical trials specifically in lymphoma patients to evaluate the efficacy and safety of thromboprophylaxis strategies guided by biomarker-driven or personalised risk models.

Lymphoma-related factors include aggressive histology (e.g., DLBCL, T-cell lymphomas), advanced stage, bulky disease, and specific sites such as CNS or mediastinal involvement. Patient-related factors include advanced age, obesity, poor performance status/immobilisation, and comorbidities. Treatment-related variables, including chemotherapy regimens, central venous catheters, and supportive agents, together with the early period after diagnosis, also increase VTE risk. APA: antiphospholipid antibodies; BEACOPP: bleomycin, etoposide, doxorubicin, cyclophosphamide, vincristine [Oncovin], procarbazine, prednisone; CNS: central nervous system; CVC: central venous catheter; DLBCL: diffuse large B-cell lymphoma; ESA: erythropoiesis-stimulating agents; FL: follicular lymphoma; G-CSF: granulocyte colony-stimulating factor; and VTE: venous thromboembolism.

## Data Availability

No new data were created or analyzed in this study. Data sharing is not applicable to this article.
